# On Some New Methods of Treating Hydrocele

**Published:** 1882-12-20

**Authors:** Robert F. Weir

**Affiliations:** Attending Surgeon to Bellevue and New York Hospitals


### ON SOME NEW METHODS OF TREATING HYDROCELE.

BY ROBERT F. WEIR, M. D.,

Attending Surgeon to Bellevue and New York Hospitals.

The usual method of treating hydrocele is by the injection of tincture of iodine into the sac of the tunica vaginalis, as suggested by Sir Ronald Martin, in 1852. This method is a very safe, and speaking generally, a successful one. Certainly it has met with the approval of the profession more than any of the other methods of injection, such as of alcohol, of port wine, sulphate of zinc, etc., and possesses advantages in regard to the small amount of inflammatory action, over the use of setons, of cutting into the hydrocele, or of removing a portion of the wall of the sac. Nevertheless, it is followed by quite a number of failures,* especially where the weakened tincture of iodine has been used, or where the surplus tincture has been allowed to flow out through the canula, as is advised by some of the British surgeons. Mr. Samuel Osborn, in a little work on hydrocele, published in 1878, found that, in fifty-four cases of the affection, treated by tapping and injection, eighteen had recurrence of the trouble. My own failures have not attained such high proportions as this, a circumstance perhaps due to the fact that not only was one to two drachms of the ordinary tincture of iodine thrown into the sac of the tunica vaginalis and there left, but also, following the suggestions made by my preceptor, the late Dr. Gurdon Buck, the inflamed scrotal tissues were, on the second or third day, rubbed and manipulated quite roughly, in order to intensify the inflammatory action, it being held by this distinguished surgeon that the injection of iodine failed by reason of too little inflammation.

Upon the advent of Listerism an old method was revived, deprived of the dangers of inflammation by the use of carbolic acid, by Professor Volkmann, of Halle, who reported, in the *Berliner Klinische Wochenscrift*, 1876, seventeen cases of hydrocele, treated by free incision of the sac under antiseptic conditions. The following are the details of the operative proceedings :

The genitals and inguinal regions are carefully and repeatedly washed with a solution of carbolic acid, and the skin of the pubic and perineal regions is closely shaved. Then, under the carbolic acid spray, the sac of the hydrocele is incised from the region of the external abdominal ring to the most dependent part of the affected side of the scrotum. The exposed and emptied cavity of the tunica vaginalis is next syringed out with a three per cent. solution of carbolic acid, and the edges of this incised membrane are stitched carefully to the corresponding edges of the incised skin by fifteen, twenty, or even more sutures of very thin silk. All bleeding vessels are ligated by fine carbolized catgut. The antiseptic dressing is applied closely and firmly, so as to maintain the inner surface of the parietal layer of the tunica vaginalis in direct contact with the surface of the corresponding testicle, a small portion of this organ, however, being left exposed between the gaping edges of the incised wound. The prolonged action of the cold carbolic acid spray on the surface of the scrotum during the application of the sutures, and the repeated injection of a cold fluid into the sac of the hydrocele, cause considerable retraction of the dartos, so that this cavity becomes much reduced in size, and its membranes closely surround the testicle. In cases where the sac is very large and lax, and also in cases where there is much fibrous induration and sclerosis, it may be necessary to excise a portion of the tunica vaginalis. The most favorable cases are those in which the extent of the sac is so far reduced by the contraction of the scrotum that a small streak of the testicular surface is left distinctly visible between the edges of the wound. The introduction of a drainage-tube is not always necessary. In some cases, however, where a portion of the tunica vaginalis forms an elongated funnel, with valvular folds on its internal surfaces, a small drainage-tube should be passed as far as the front of the testicle. The blood having been removed from the seat of operation, the scrotum is surrounded by eight or nine strips of antiseptic gauze, and a large piece of gauze, in eight layers, with a corresponding slit in each layer for the passage of the penis, is placed over theze. This dressing is closely maintained in its place by a bandage of gause saturated with carbolic acid. All gaps in the dressing are stopped with portions of salicylized wool, and a large pad of this material is placed in the perineum, between the anus and the scrotum. Absolute antiseptic sealing of the wound is a necessary condition of total occlusion of the sac. On the first change of the dressing, the cavity will be found to be quite occluded, and the wall of the hydrocele-cavity closely adherent to the surface of the testicle."

These seventeen cases were subsequently increased to sixty-nine in a report of Dr. Genzmer in Volkmann's Klinische Vortrager, No. 135. In none of these was there any excessive inflammation, and the average duration of the stay of the patient in the hospital was ten days. The tube, if any was used, was removed generally about the fourth day, when the silk sutures were also taken out, and the dressing changed a second time at the end of a week. The wound was then dressed with the salicylized cotton batting inside of a suspension bandage, and the patient discharged.

I can corroborate the satisfactory progress of patients treated by this method. I have now resorted to this procedure some twenty-eight times, and in but two instances they have all proceeded as satisfactorily as above indicated by Volkmánn. In these two, through error of manipulation on the part of my house surgeon, who performed the operation, there was two much stripping up of the skin from the subjacent tissue and some slight sloughing took place. The dressings were removed on an average about the eighth or tenth day. To promote the final healing of the little linear wound which was then left, a solution of nitrate of silver, five grains to the ounce, was painted daily on the granulations, as has been suggested by one of the German surgeons. This method has also been used with satisfaction in two cases of hæmatocele.

On reasoning out this process it is evident that after the evacuation of the fluid of the hydrocele, the flushing of the serous membrane by a solution of carbolic acid, the drainage-tube and the antiseptic dressing are the essentials of the operation. Now, in the hope of avoiding an extensive incision, I subsequently modified this procedure of Volkmann in the following way: the hydrocele was evacuated by a large size trocar and through the canula, held in situ, a solution of one of the fifteen carbolic solution (adding a small quantity of glycerine to perfect the solution of carbolic acid) was injected so as to wash out the cavity thoroughly. A small, rubber drainage-tube was then introduced through the same canula before the latter was finally removed. This was done without ether, but under the antiseptic spray and other precautions. The usual Lister dressing was then applied, carbolized jute being substituted for the salicylated cotton in use in Europe. The drainage-tube was removed on the third, fourth and fifth day, when the dressing was changed, and at the end of a week the antiseptic precautions were abandoned. My experience embraces some ten† cases in which this has been performed with satisfactory results, and in only one was there any inflammatory action, which was due to the patient's persistent disturbance of the antiseptic dressings. Though this plan was an improvement upon Volkmann's operation, it was yet hampered by the necessity of the use of the spray and antiseptic dressings. The fact, however, became patent, by resorting to these injections that they were free from pain, and I was, therefore, prepared to accept a statement that came to my notice in the Philadelphia Medical Times of the issue of November 6, 1880, by Dr. J. R. Levis, who advised that hydrocele be treated by the injection of a small quantity of pure carbolic acid into the sac of the tunica vaginalis, a procedure that he stated he had used since 1872. His method is to withdraw the fluid by an ordinary trocar, and then to introduce the long, slender nozzle of a syringe through the canula into the vaginal sac. By this means the carbolic acid is readily thrown into the serous cavity, and there is no danger of its being injected into the cellular tissue of the scrotum. The carbolic crystals are liquefied by slight heat or by the addition of a few drops of glycerine. The amount of carbolic acid which Dr. Levis injects is one-half a fluid drachm, and this is allowed to remain permanently in contact with the tunica vaginalis. The operation, he further states, is almost, if not entirely, painless, because of the local anæsthetic action of the carbolic acid. Patients sometimes exclaim at the moment of the introduction, but complain of a sense of numbness, rather than of pain. The pain is certainly much less than where tincture of iodine is employed. Care, however, should be observed to allow no acid to flow upon the surface of the scrotum, for pain and inflammation will follow such contact. Dr. Levis had never seen suppuration or sloughing follow this manner of dealing with hydrocele.

I was led, during the early portion of last winter, to adopt this mode of treatment, and though prepared by my previous experience to believe that the carbolic injections were not very irritating, yet I was most agreeably surprised to find that the strong acid was, in the instances which have come under my notice, absolutely painless, or so very slightly so as to be insignificant. In my services at Bellevue Hospital, as well as in private practice, I have used this injection in thirteen patients, four of whom had double hydrocele, with only one failure. In the male hydrocele both sacs were injected at the same sitting. In this instance there was reaccumulation, and a second injection of the pure carbolic acid, two weeks later, was followed by a cure. In this action I afterward learned how to precipitate, for the fluid, when reformed, will gradually disappear, as after an iodine injection. In a majority of instances there was not such a reaccumulation of fluid, but there was considerable thickening of the whole tunica vaginalis, both parietal and visceral. This thickening will last three or four weeks, sometimes longer Patients are at no time incapacitated from attending to their usual vocations, though Levis speaks of their being detained for a day at most from such. In every instance the urine was carefully tested for three or four days subsequent to the operation, in order to determine whether or not there had been absorption of carbolic acid. None was observed in my case. The quantity injected was one-half to one and one-half drachm.

I feel warranted, therefore, from this experience, in corroborating this favorable judgment passed upon this method of treatment by its promulgator, Dr. Levis, and in presenting it as a procedure safe and painless, and, so far as my limited number of cases go, effectual in its results.—*N. Y. Med. Record.*

---

* Erichesen says: "Useful as the iodine injection is, it sometimes fails in producing a radical cure of hydrocele," and again, "it is by no means improbable that the success of the iodine injection in this country might not prove to be quite so great as is generally believed."—(Syst. of Surgery.)

Curling says: "Iodine injection is not capable of affecting a cure in every case." He also admits the painfulness of the injection which is quite frequently encountered, and suggests the use of an anæsthetic.—(Diseases of Tests.)

Agnew believes that the failures, when they occur, are due to the wrong use of the injection. The undiluted tincture of iodine should be thrown in and allowed to remain.—Princ. and Pract. of Surgery, vol. ii.)

† Six of these have been already reported in the New York Medical Journal, December, 1880, p. 631.

